# Evaluation of a Short Message Service (SMS)-Based Health Education Program (“Active Moms”) on Antenatal Knowledge and Health Attitudes Among Primigravida Women in South Gujarat: A One-Group Pretest-Posttest Study

**DOI:** 10.7759/cureus.100013

**Published:** 2025-12-24

**Authors:** Pooja Parmar, Sajidali S Saiyad, Grishma Chavda, Gnanadesigan Ekambaram, Jay Prakash S Rajput

**Affiliations:** 1 Obstetrics and Gynaecology, J and D Institute of Nursing, Surat, IND; 2 Physiology, Pacific Medical College and Hospital, Pacific Medical University, Udaipur, IND; 3 Obstetrics and Gynaecology, Dr. N. D. Desai Faculty of Medical Science and Research, Dharmsinh Desai University of Nadiad, Nadiad, IND; 4 Physiology, Nootan Medical College and Research Centre, Sankalchand Patel University, Visnagar, IND; 5 Physiology, Shri Gorakshnath Medical College Hospital and Research Centre, Gorakhpur, IND

**Keywords:** antenatal care (anc), behavior change communication (bcc), digital health intervention, health attitude improvement, india public health innovation, low- and middle-income countries (lmics), maternal health literacy, mobile health (mhealth), primigravida women, sms-based health education

## Abstract

Introduction

Maternal health education during antenatal care remains suboptimal among primigravida women, particularly in semi-urban and rural settings, leading to gaps in knowledge and health-related attitudes. Short message service (SMS)-based interventions offer a low-cost and accessible strategy to address these gaps by reinforcing essential antenatal information.

Methods

A quantitative one-group pretest-posttest design was implemented among 60 primigravida women in South Gujarat. Participants received standardized SMS messages on antenatal topics, nutrition, hygiene, rest, supplementation, danger-sign recognition, and delivery preparedness, adapted from WHO guidelines and localized in Gujarati. Data were collected using validated instruments assessing knowledge (20 items) and attitudes (22-item Likert scale). Paired-samples t-tests were used to analyze changes in knowledge and attitude scores, while chi-square tests and Pearson’s correlation assessed demographic associations and relationships between outcomes. Paired t-tests, chi-square tests, and correlation analyses were conducted using SPSS v25 (IBM Corp., Armonk, NY, USA), with significance set at p < 0.05.

Results

Mean knowledge scores increased significantly from 12.0 ± 2.4 to 16.8 ± 1.9 (p < 0.001, Cohen’s d = 1.30), while mean attitude scores rose from 16.0 ± 1.17 to 18.6 ± 1.0 (p < 0.001, d = 1.10). A strong positive correlation was observed between knowledge and attitudes (r = 0.62, p < 0.001). Education level, nuclear family type, and higher income were significantly associated with greater knowledge gain. The intervention accounted for 42% of the variance in outcomes (η² = 0.42).

Significance

The Active Moms SMS-based intervention was associated with improvements in antenatal knowledge and health attitudes among primigravida women. Given the exploratory nature of this one-group pretest-posttest study and the absence of a control group, these findings should be interpreted cautiously. Nonetheless, the results suggest that SMS-based education may represent a promising, scalable, culturally adaptable, and cost-effective approach to supporting maternal health education in low-resource settings. Further controlled studies are needed to confirm effectiveness before large-scale integration into national antenatal care frameworks.

## Introduction

Maternal health is a global public health priority, with antenatal care (ANC) serving as a cornerstone for reducing maternal and neonatal morbidity [1]. Regular ANC facilitates early identification of complications, promotes healthy behaviors, and enhances maternal preparedness for delivery. However, in low- and middle-income countries (LMICs), especially in India, disparities in knowledge and attitudes toward antenatal care remain prominent [2]. Although institutional deliveries and antenatal care (ANC) coverage have improved, evidence from national surveys and regional data from Gujarat and western India indicates that many women, particularly first-time mothers in semi-urban and rural areas, continue to lack comprehensive understanding of nutrition, hygiene, and danger-sign recognition during pregnancy [3]. These gaps highlight the urgent need for innovative, accessible strategies to improve maternal awareness and engagement.

With the rapid penetration of mobile technology, digital health interventions have emerged as effective tools to strengthen communication between health systems and beneficiaries [4]. The World Health Organization defines mobile health (mHealth) as the use of mobile and wireless technologies to support public health objectives [5]. Short message service (SMS)-based programs are particularly advantageous due to their simplicity, low cost, and ability to reach large populations compared with smartphone applications or internet-dependent mHealth platforms, as they do not require smartphones, continuous data access, or advanced digital literacy. Evidence from multiple LMICs, including Bangladesh, Kenya, and Tanzania, suggests that tailored text messages can improve ANC attendance and iron-folic acid adherence. These findings highlight the potential of mobile health (mHealth), the use of mobile and wireless technologies such as SMS, voice calls, and mobile applications for health promotion, to support maternal behavior change [6].

In India, several large-scale initiatives such as Kilkari and Mobile Academy have demonstrated the feasibility of mobile communication for maternal health promotion. However, these programs primarily focus on service utilization and awareness at a population level, with limited evaluation of changes in maternal knowledge and health attitudes, particularly among primigravida women. The present study addresses this gap by evaluating a localized, SMS-only intervention and its association with both cognitive and attitudinal outcomes in a semi-urban Indian setting. However, most initiatives rely on one-way communication and have not adequately evaluated changes in maternal attitudes. Evidence from recent studies suggests that while such voice- or SMS-based programs can enhance maternal practices, particularly in comparison with smartphone applications or internet-dependent mHealth platforms, SMS does not require smartphones, continuous internet access, or advanced digital literacy. For example, the mMitra trial in India demonstrated improvements in immunization and institutional delivery rates but reported modest changes in maternal health beliefs and self-confidence [7]. Similarly, a scoping review of perinatal mHealth programs in India found that most interventions relied on unidirectional text messages and rarely evaluated psychological or attitudinal constructs, underscoring a gap in understanding how digital communication influences women’s motivation and empowerment [8]. Systematic reviews confirm that SMS interventions improve service uptake, but their effect on maternal attitudes remains unclear [9]. Global meta-analyses of pregnancy-related behavioral interventions also report that self-efficacy outcomes are infrequently measured and inconsistently defined [10]. In contrast, smaller studies using interactive or two-way phone calls have shown promising effects on women’s perceived competence in antenatal care [11]. However, there remains limited evidence from western India, particularly South Gujarat, where literacy levels, cultural norms, and clinic workload constraints may hinder effective counseling, especially for primigravida women who require structured, trimester-specific education.

The present study was conceptualized to address this evidence gap through a pre-experimental evaluation of the “Active Moms” SMS-based health education program. Grounded in Imogene M. King’s Theory of Goal Attainment, the intervention uses concise, evidence-based messages to promote informed decision-making and positive behavioral change among pregnant women. The study aimed to assess the effectiveness of this program in improving antenatal knowledge and health attitudes among primigravida women in South Gujarat and to explore associations with selected demographic variables. By providing region-specific data, the findings are expected to inform future integration of SMS-based education within India’s maternal health framework, offering a scalable, low-cost strategy to enhance antenatal literacy and empower women toward safer motherhood. Accordingly, this study aimed to evaluate the association of an SMS-based health education program (“Active Moms”) with changes in antenatal knowledge and health attitudes among primigravida women in South Gujarat. Secondary objectives included examining the relationship between selected sociodemographic factors (education level, family type, and household income) and antenatal knowledge levels, as well as assessing the correlation between antenatal knowledge and health attitudes following the intervention.

## Materials and methods

Study design and setting

A quantitative one-group pretest-posttest design was adopted to evaluate changes in antenatal knowledge and health attitudes following the “Active Moms” SMS-based education program. The same group of participants was assessed twice, before the first SMS exposure (pretest) and two weeks after completion of the intervention period (posttest). 

This design was selected to assess short-term educational outcomes in a real-world clinical setting where randomized controlled comparisons were not feasible due to resource and ethical constraints. The study was conducted in selected hospitals in South Gujarat, India, which provide routine antenatal care services, including outpatient ANC visits and health education counseling.

Participants and sampling

The study population comprised primigravida women in their second trimester (13-27 weeks of gestation) attending antenatal clinics during February 2020. The second trimester was selected because it is a stable phase of pregnancy, during which most women have initiated antenatal care, experience fewer early-pregnancy symptoms, and have adequate time for health education interventions to influence antenatal practices, supplementation adherence, and danger-sign recognition before delivery. Eligible participants were those able to read Gujarati or English and willing to provide informed consent. Women outside the second trimester or unwilling to participate were excluded. Baseline (pre-intervention) assessments of knowledge and attitudes were obtained before sending the first set of SMS messages, and the same instruments were re-administered two weeks after program completion. A non-probability purposive sampling technique with consecutive recruitment was used, whereby all eligible primigravida women attending the outpatient antenatal departments during the study period were approached sequentially until the required sample size was reached.

Sample-size determination

Based on prior mHealth education studies reporting moderate pre-post improvements in antenatal knowledge scores, the expected mean improvement (Δ) was conservatively assumed to be 2 points, with a standard deviation (σ) of 3.5 points. A moderate pre-post correlation coefficient (r = 0.5) was assumed for the paired design, as the same validated instrument was administered before and after the intervention. Such moderate correlations are commonly reported and recommended in sample-size planning for educational and behavioral interventions when prior empirical correlation estimates are unavailable, and they provide a conservative balance between independence and high correlation assumptions [5,9].

The sample size was estimated using the formula for a paired-sample t-test:

\[ n = \frac{(Z_{1-\alpha/2} + Z_{1-\beta})^2 \, \sigma_d^2}{\Delta^2} \]

where $Z_{1-\alpha/2}$ = 1.96 (two-tailed α = 0.05), $Z_{1-\beta}$ = 0.84 (power = 80%), $\sigma_d$ = standard deviation of the difference, and $\Delta$ = expected mean improvement.

Based on prior mHealth education studies, the expected mean improvement $\Delta$ in knowledge score was assumed to be 2 points with a standard deviation (σ) of 3.5 points and a pre-post correlation (r) of 0.5, giving $\sigma_d$ = ${2\sigma^2(1-r)}$ = 3.5.

Substituting these values:

\[ n = \frac{(1.96 + 0.84)^2 \times (3.5)^2}{(2)^2} = 24.01 \]

Allowing for a 20% attrition rate and ensuring adequate power for secondary binary outcomes (improvement in “adequate knowledge” category, expected net change ≈ 25%), the final required sample was ≈ 57, rounded to 60 participants. This ensured >80% power to detect a moderate effect (Cohen’s d ≈ 0.5).

Intervention

The Active Moms program consisted of 15 standardized SMS messages developed from WHO Antenatal Care (2016) guidelines and reviewed by a panel of five obstetric and public-health experts for content validity (Content Validity Index (CVI) = 0.89). Messages covered nutrition (three), personal hygiene (two), rest and physical activity (two), supplement adherence (three), danger-sign recognition (three), and birth preparedness (two). Each message was limited to 160 characters, translated into Gujarati, and delivered once daily for 15 consecutive days. Comprehension was checked through verbal feedback during follow-up ANC visits.

Instruments and Data Collection

Three instruments were used: (a) Demographic Proforma; (b) Structured Knowledge Questionnaire (20 multiple-choice items, score 0-20); and (c) Antenatal Health Attitude Scale (22-item Likert items, score 22-66). Both tools were adapted from Indira et al. (2017) and refined after expert review [5]. Pilot testing on 10 primigravida women yielded Cronbach’s α = 0.83 (knowledge) and 0.86 (attitude), indicating good internal consistency. Content Validity Index (CVI) was 0.89. Readability was tested using the Flesch-Kincaid Grade 7 equivalent in Gujarati translation. Copies of final tools are included in Appendix A-C.

The instruments used in this study were either self-developed or adapted from publicly available academic literature (Indira et al., 2017) and WHO ANC guidelines. No standardized or proprietary scales were utilized, and the adapted items do not contain copyrighted material; hence, formal permission was not required.

Baseline (pre-intervention) assessments of antenatal knowledge and attitudes were conducted prior to SMS delivery using the same standardized instruments. Post-intervention data were collected two weeks after completion of the message sequence from the same participants. Although randomized control was not feasible in the given setting, the pretest-posttest design ensured internal comparison within the same participants, minimizing inter-subject variability. This design is widely used in preliminary evaluations of behavioral interventions in low-resource environments.

Data analysis

Effect sizes were calculated as Cohen’s d for paired data using the mean difference divided by the standard deviation of differences. Values > 0.8 were interpreted as large effects, acknowledging possible inflation due to same-sample design and small N. The partial eta-squared (η²) was derived from the paired t-test using η² = t² / (t² + df) to represent the proportion of variance explained by the intervention.

Knowledge and attitude scores were categorized as inadequate (<50%), moderate (50%-79%), and adequate (≥80%) following standard educational classification (Polit and Beck, 2021) [12]. This enabled chi-square testing of associations with demographic variables. Pre-intervention and post-intervention scores were compared using paired-samples t-tests to evaluate within-group change.

Baseline sociodemographic characteristics were examined descriptively in relation to pre-intervention knowledge and attitude scores. As the study employed a single-group pretest-posttest design, the primary analysis focused on within-participant change rather than baseline subgroup comparisons. Descriptive statistics (frequency, percentage, mean ± SD) were used to summarize demographic characteristics and baseline variables. Paired-samples t-tests were applied to compare pre- and post-intervention knowledge and health-attitude scores. Pearson’s correlation coefficient was used to assess the relationship between post-intervention knowledge and attitude scores. Chi-square tests were employed to examine associations between categorized knowledge levels and selected demographic variables. Effect sizes were calculated using Cohen’s d for paired data, and partial eta-squared (η²) was computed to estimate the proportion of variance attributable to the intervention. Statistical significance was set at p < 0.05.

Ethical considerations

Ethical approval and administrative permissions were obtained from the Ethics Committee of the Unique Hospital Multispecialty and Research, Surat, India (Dated 26-01-2020). Participants were informed about study objectives, assured of confidentiality, and provided written consent before inclusion.

## Results

Participant characteristics

A total of 60 primigravida women completed the pre-experimental study evaluating the Active Moms SMS-based health-education intervention. Baseline sociodemographic data are summarized in Table 1. Most participants, 40 (66.7%), were between 18 and 22 years of age, while 20 (33.3%) were 23 to 28 years old. Educational attainment was predominantly at the secondary level, 42 (70.0%), followed by primary, 14 (23.3%), and graduation, 4 (6.7%). The majority, 53 (88.3%), identified as Hindu, whereas seven (11.7%) were Muslim. Slightly more than half, 33 (55.0%), lived in nuclear families, and 27 (45.0%) belonged to joint families. Nearly half, 28 (46.7%), reported a monthly household income below ₹ 5,000, 23 (38.3%) earned between ₹ 5,001-10,000, and nine (15.0%) fell within ₹ 10,001-15,000.

**Table 1:** Baseline demographic characteristics of participants (N = 60) Data are presented as frequency (n) and percentage (%). Descriptive statistics were used to summarize participant characteristics. No inferential test was applied.

Variable	Category	n (%)
Age (years)	18-22	40 (66.7)
	23-28	20 (33.3)
	≥29	0 (0)
Education	Illiterate	0 (0)
	Primary	14 (23.3)
	Secondary	42 (70.0)
	Graduate	4 (6.7)
Religion	Hindu	53 (88.3)
	Muslim	7 (11.7)
Family type	Joint	27 (45.0)
	Nuclear	33 (55.0)
Monthly income (₹)	< 5,000	28 (46.7)
	5,001-10,000	23 (38.3)
	10,001-15,000	9 (15.0)
	> 15,000	0 (0)

Baseline distribution of knowledge and attitude levels is presented in Table 2. 

**Table 2:** Baseline distribution of knowledge and attitude categories (pre-intervention, N = 60) Descriptive baseline distribution of participants’ antenatal care knowledge and attitudes before the intervention.

Variable	Inadequate n (%)	Moderate n (%)	Adequate n (%)	Mean ± SD (%)
Knowledge	10 (16.7%)	50 (83.3%)	0 (0%)	12.0 ± 2.4 (60.0%)
Attitude	0 (0%)	30 (50%)	30 (50%)	16.0 ± 1.17 (72.7%)

Knowledge scores on antenatal care

Pre-intervention assessments revealed moderate baseline awareness of antenatal care. Following exposure to the Active Moms SMS program, post-test scores increased significantly. For general aspects (six items), the mean score was 4 ± 1.1 (66.7%); for physical changes (seven items) 4 ± 1.2 (57.1%); and for diet-related items 4 ± 1.3 (57.1%). The overall pre-intervention knowledge mean was 12 ± 2.4 (out of 20; 60%). Following exposure to the Active Moms SMS program, the mean post-intervention score increased significantly to 16.8 ± 1.9 (84%). The mean difference of 4.8 points (95% CI: 2.9-5.5, p < 0.001) confirmed a statistically significant improvement. The calculated Cohen’s d = 1.30 demonstrated a large effect of the intervention on knowledge acquisition. The detailed comparison of pre- and post-intervention knowledge scores is shown in Table 3.

**Table 3:** Comparison of pre- and post-intervention knowledge scores (N = 60) Data are expressed as Mean ± SD, 95% Confidence Interval (CI), t-value, and p-value. The paired-samples t-test was used to assess differences in mean knowledge scores before and after the intervention. A p-value < 0.05 was considered statistically significant; p < 0.001 was considered highly significant. Statistical parameters: Mean ± SD, 95% CI, t, p, and Cohen’s d (effect size). *** denotes high statistical significance (p < 0.001).

Parameter	Mean ± SD	Mean %	95% CI	p-value	Cohen’s d
Pre-intervention	12.0 ± 2.4	60.0	—	—	—
Post-intervention	16.8 ± 1.9	84.0	2.9-5.5	<0.001 ***	1.30

Although the observed Cohen’s d value indicates a large magnitude of change, this estimate should be interpreted cautiously, as one-group pretest-posttest designs are susceptible to effect-size inflation due to repeated testing and the absence of a control group. Most women initially demonstrated only moderate awareness, but the majority achieved adequate knowledge after receiving the SMS messages, reflecting strong educational impact.

Health attitude scores

Attitudinal responses mirrored the improvement seen in knowledge outcomes. Participants demonstrated more positive attitudes toward antenatal care after the intervention, with most women moving from average to good attitude levels. This reflects meaningful reinforcement of confidence and motivation through the SMS messages. The computed Cohen’s d = 1.10 indicated another large effect size. Prior to intervention, equal proportions of participants 30 (50%) demonstrated average and good attitudes; following the program, 51 (85%) reported good attitudes. Changes in antenatal health-attitude scores are presented in Table 4.

**Table 4:** Comparison of pre- and post-intervention health-attitude scores (N = 60) Data are presented as Mean ± SD, 95% CI, t-value, and p-value. A paired-samples t-test was employed to compare mean health-attitude scores before and after intervention. A p-value < 0.05 denoted significance; p < 0.001 denoted high significance. *** denotes high statistical significance (p < 0.001).

Parameter	Mean ± SD	Mean %	95% CI	p-value	Cohen’s d
Pre-intervention	16.0 ± 1.17	72.7	—	—	—
Post-intervention	18.6 ± 1.0	84.5	1.9-3.1	< 0.001***	1.10

Similarly, the large effect size observed for health-attitude scores should be interpreted with caution, as repeated measurement and lack of a comparison group may contribute to overestimation of effect magnitude in one-group pretest-posttest designs.

Correlation between knowledge and health attitudes

**Figure 1:**
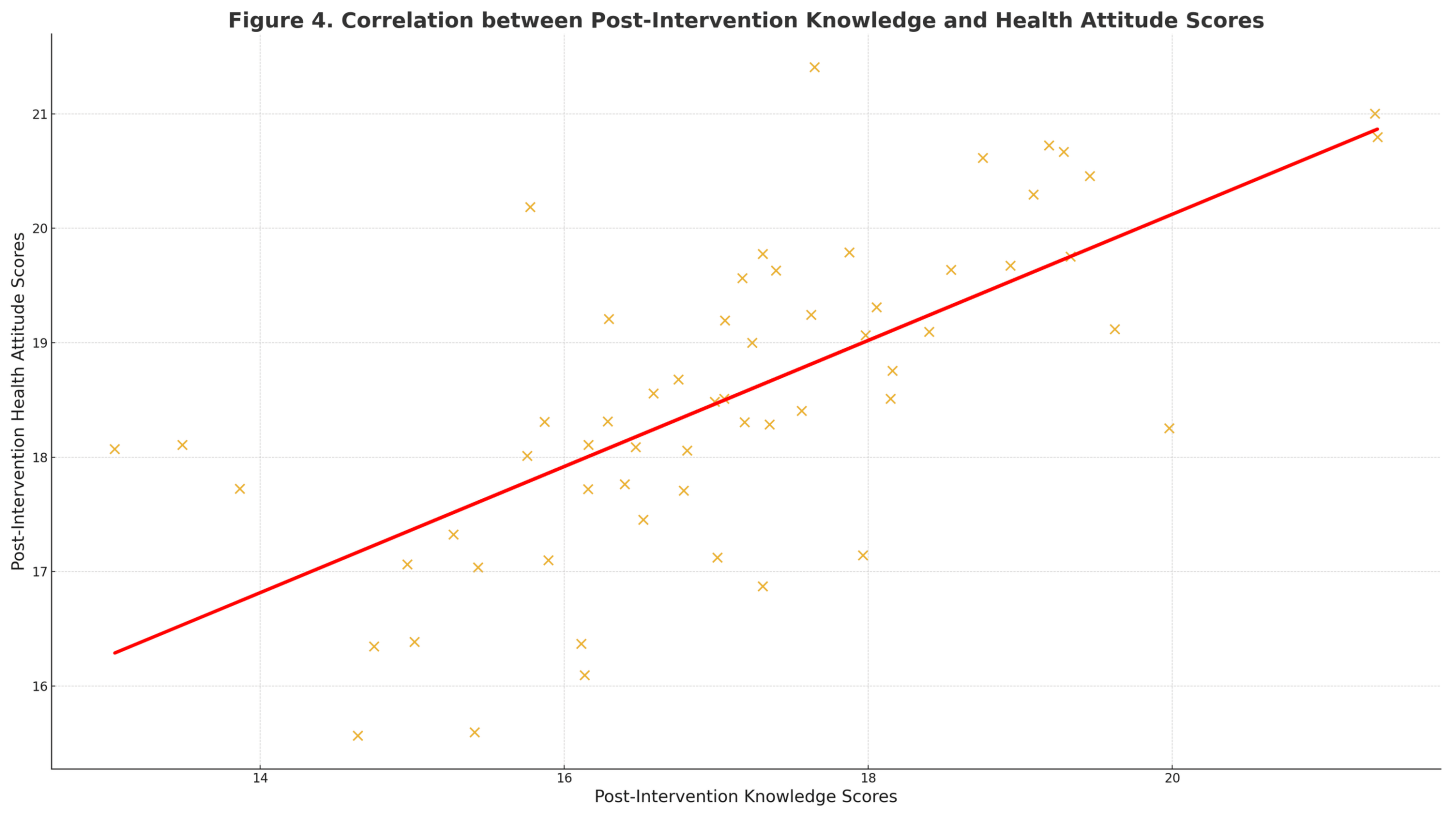
Scatter plot showing correlation between post-intervention knowledge and attitude scores Scatter plot showing the linear correlation (r = 0.62, p < 0.001) between post-intervention knowledge and health-attitude scores among primigravida women. Regression line indicates positive correlation. Data represented as individual participant scores (continuous variables). Statistical test: Pearson’s correlation coefficient (r). Significance: p < 0.05 considered significant; p < 0.001 highly significant.

Knowledge and attitude scores were positively related, indicating that women who learned more also developed more favorable health attitudes. Participants who attained higher knowledge also tended to report more favorable health attitudes toward antenatal care, confirming internal consistency between cognitive and affective learning domains. The correlation between post-intervention knowledge and attitude scores is illustrated in Figure 1.

Association between demographic variables and knowledge

**Figure 2:**
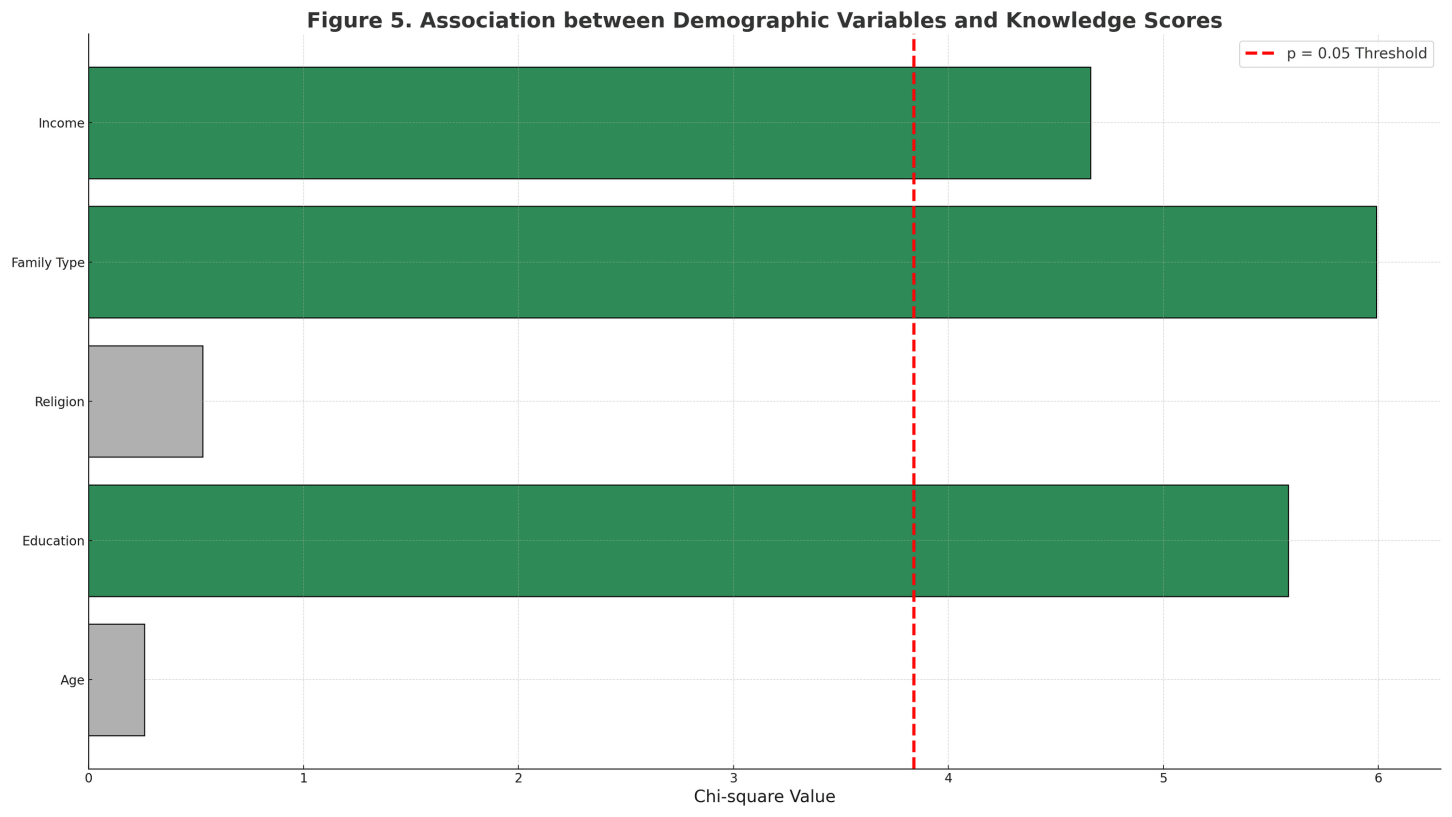
Forest plot illustrating chi-square associations for demographic predictors Forest-style horizontal bar plot illustrating chi-square values for demographic predictors of knowledge levels (age, education, religion, family type, and monthly income). The red dashed line (χ² = 3.84) denotes the p = 0.05 significance threshold. Bars in green represent significant associations (p < 0.05), while grey bars indicate non-significant results (p > 0.05). Statistical test: Pearson chi-square test.

Associations between key demographic characteristics and knowledge levels were examined using chi-square tests (Table 5). The chi-square associations for demographic predictors are displayed in Figure 2. These findings suggest that higher educational attainment, nuclear family structure, and better income levels contributed to greater knowledge gain following the intervention.

**Table 5:** Association between demographic variables and knowledge levels (N = 60) Data are expressed as χ² (chi-square value) and p-value. Associations were examined using the Pearson chi-square test. A p-value < 0.05 was considered statistically significant.

Variable	χ²	p-value	Significance
Age	0.26	0.61	Not significant
Education	5.58	0.02	Significant
Religion	0.53	0.47	Not significant
Family type	5.99	0.01	Significant
Monthly income	4.66	0.03	Significant

Effect size summary

Quantitative synthesis of intervention effects demonstrated large pre-post changes in both outcome domains. For antenatal care knowledge, the standardized mean difference was large (Cohen’s d = 1.30), while health-attitude scores also showed a large effect (Cohen’s d = 1.10). For antenatal knowledge scores, the paired-samples t-test yielded a partial eta-squared (η²) of 0.42, indicating that approximately 42% of the variance in pre-post knowledge scores was associated with the SMS-based educational intervention. Effect sizes for health-attitude outcomes were therefore summarized using Cohen’s d.

However, these effect-size estimates should be interpreted with caution, as one-group pretest-posttest designs are susceptible to effect-size inflation due to repeated testing and the absence of a control group. Accordingly, the observed magnitudes should be viewed as indicators of potential intervention impact rather than definitive estimates of efficacy.

Summary of findings

The participants were predominantly young, secondary-educated, low-income women residing in South Gujarat. Baseline assessments revealed moderate knowledge and attitudes regarding antenatal care. Following exposure to the structured SMS-based education program, statistically significant and practically large improvements were observed in both knowledge and attitude domains (p < 0.001). Education, family type, and income significantly influenced knowledge gains, echoing earlier observations that literacy, household autonomy, and socioeconomic support affect women’s ability to act on health information [9]. Together, these results affirm the effectiveness of the Active Moms SMS intervention in enhancing antenatal awareness and promoting favorable maternal health behaviors among primigravida women in the region.

## Discussion

The present pre-experimental pretest-posttest study evaluated the short-term effectiveness of an SMS-based health education program, Active Moms, designed to improve antenatal knowledge and health attitudes among primigravida women in South Gujarat. Exposure to SMS messages was associated with higher knowledge scores and more positive health attitudes within the study group; however, in the absence of a comparison group, these improvements cannot be attributed solely to the intervention and should be interpreted as exploratory associations. Although the design does not permit causal inference, the findings suggest the potential effectiveness of SMS-based education in this population. These results highlight the potential of short, culturally adapted text messages to enhance maternal health literacy and engagement with antenatal care (ANC) services, particularly in low-resource settings.

Interpretation of principal findings

The observed improvements from baseline to post-intervention confirm that measurable learning occurred within the same participant group following exposure to the SMS-based education program. The significant increase in knowledge scores (60% to 84%) and health-attitude scores (73% to 85%) suggests that even brief, unidirectional SMS reinforcement can support meaningful learning and behavioral readiness among primigravida women. Repeated and concise messaging likely facilitated information recall and reinforced positive health orientations. This finding aligns with the Health Belief Model, which posits that exposure to relevant cues enhances perceived benefits and reduces barriers to action, thereby promoting health-related behavior change (Ensor and Cooper [13]). The strong positive correlation observed between post-intervention knowledge and attitude scores (r = 0.62) further indicates that cognitive gains were accompanied by affective change, an essential determinant of sustained antenatal care adherence.

Sociodemographic factors such as education level, family type, and household income significantly influenced knowledge gains, corroborating prior evidence that literacy, household autonomy, and socioeconomic support shape women’s capacity to access and apply health information (Bitew et al. [14]; Wagnew et al. [9]). The greater benefit observed among women from nuclear families suggests that SMS-based interventions may partially compensate for the limited interpersonal guidance traditionally available in joint-family settings, particularly for first-time mothers. Although the large effect sizes observed for both knowledge and health-attitude outcomes indicate substantial pre-post improvements, these estimates should be interpreted with caution. In one-group pretest-posttest designs, effect sizes may be inflated due to repeated testing, increased familiarity with questionnaire items, regression to the mean, and the absence of a control group. Accordingly, the reported effect sizes should be viewed as indicators of potential intervention impact rather than definitive measures of efficacy, underscoring the need for controlled studies to confirm the magnitude and sustainability of observed effects.

Beyond statistical significance, the observed 4.8-point increase in antenatal knowledge scores reflects meaningful gains in participants’ understanding of key ANC domains, including maternal nutrition, iron-folic acid supplementation, recognition of pregnancy danger signs, and the importance of timely healthcare seeking. Although behavioral practices and clinical outcomes were not assessed, such improvements in knowledge and attitudes represent essential enabling prerequisites for informed decision-making during pregnancy and may support improved maternal self-care when integrated with routine antenatal counseling.

Comparison with previous studies

The present findings are congruent with prior mHealth interventions reporting improvements in maternal knowledge and antenatal care (ANC) utilization. In India, initiatives such as the Kilkari voice-messaging program and other interactive mHealth interventions have demonstrated positive effects on maternal awareness and service uptake. However, it is important to note that many of these studies employed randomized or controlled designs, which differ methodologically from the one-group pretest-posttest design used in the present study. Consequently, effect sizes and magnitudes of change are not directly comparable across study designs, and the similarities observed should be interpreted as directional consistency rather than equivalence of impact. In India, the Kilkari voice-messaging initiative showed enhanced awareness of iron-folic acid supplementation and birth preparedness (Nimmagadda et al. [15]). Similarly, an SMS program in Maharashtra significantly increased compliance with antenatal visits and micronutrient intake (Singh et al. [16]). Our results strengthen this evidence by focusing on first-time mothers and showing large cognitive and attitudinal gains through text-only delivery.

It is important to note that the present findings should be interpreted as exploratory, given the one-group pretest-posttest design. Effect sizes observed in this study are not directly comparable to those reported in randomized or interactive mHealth trials, as pre-experimental designs may overestimate magnitude due to testing effects and the absence of a comparison group. Accordingly, similarities with prior studies should be interpreted as directional consistency rather than equivalence of impact.

Similar improvements in ANC knowledge have been reported in African and Southeast Asian SMS programs [17]. Bakibinga et al. [18] in Kenya and Oyeyemi and Wynn [19] in Nigeria likewise observed significant increases in maternal awareness and facility deliveries following SMS education. Studies in Indonesia and Myanmar found similar patterns for diet diversity and pregnancy-danger-sign recognition (Hmone et al. [20]; Tobe et al. [21]). The present study aligns with these international findings, confirming that SMS interventions transcend geographic and cultural boundaries when locally adapted.

Nevertheless, certain distinctions are noteworthy. Whereas some African programs used interactive (two-way) platforms permitting real-time queries (Kazi et al. [22]), Active Moms was unidirectional, yet it achieved large effects. This suggests that personalization, linguistic localization, and cultural relevance, rather than interactivity alone, drive message efficacy. Delivering content in Gujarati with examples grounded in daily routines likely enhanced comprehension, as previously observed in localized mHealth trials in Rwanda (Ngabo et al. [23]) and Ghana (Laar et al. [24]).

Theoretical and behavioral implications

The results may be interpreted through the lens of social-cognitive theory and the concept of self-efficacy. Continuous exposure to credible, actionable information enhances women’s confidence to undertake healthy behaviors (AlJaberi [25]). By reiterating essential ANC components, balanced diet, rest, immunization, and early recognition of complications, Active Moms reinforced self-efficacy and outcome expectancy. Furthermore, text messages serve as external cues that sustain motivation beyond the clinical setting, bridging the gap between knowledge acquisition and practical adherence (Ali Alahmari et al. [26]).

From a pragmatic standpoint, these findings validate SMS as a low-cost, scalable, and equitable communication tool. In contrast to smartphone-dependent applications, text messaging functions on basic mobile phones, ensuring inclusivity among socioeconomically disadvantaged groups (Mechael and Batavia [27]). The simplicity and asynchronous delivery reduce dependence on health-worker time while maintaining fidelity of information transfer. Such scalability is particularly valuable in India, where the ratio of Accredited Social Health Activist (ASHA) workers to pregnant women remains sub-optimal.

Alignment with national and global strategies

The observed effectiveness of Active Moms supports India’s broader digital-health agenda under the National Digital Health Mission and complements existing platforms such as Kilkari and Mobile Academy. While these national programs primarily disseminate voice content, text-based models permit easier translation, scheduling flexibility, and potential integration with electronic maternal records. Evidence from other Indian trials (Nimmagadda et al. [15]; Singh et al. [16]) indicates that combining voice and text messaging yields synergistic effects; hence, policy integration of bilingual or multimodal messaging could further optimize outcomes.

Globally, the findings align with WHO’s mHealth guidelines recommending SMS reminders and educational messages to improve maternal-health coverage in low- and middle-income countries (Bakibinga et al. [18]). Empirical data from Kenya (Kazi et al. [22]), Malaysia (Temple et al. [28]), and Bangladesh (Tobe et al. [21]) confirm SMS efficiency in increasing ANC attendance, supplementation adherence, and knowledge of warning signs. The convergence of these results underscores that text-based interventions represent a universally adaptable, evidence-backed modality for strengthening ANC engagement.

Public-health and policy implications

The demonstrated success of Active Moms offers a practical template for integration into India’s public-health infrastructure. Primary Health Centers could enroll expectant mothers at the first ANC visit and schedule automated, stage-appropriate SMS sequences. Messages can be aligned with national ANC guidelines, ensuring standardization across districts. Given that message delivery costs are minimal (< ₹10 per beneficiary per month), the program could be sustained within existing Reproductive and Child Health budgets.

At a system level, the intervention aligns with the Sustainable Development Goal 3 target of reducing maternal mortality to below 70 per 100,000 live births by 2030. Improved awareness of nutrition, rest, and warning signs could translate into earlier help-seeking, timely referrals, and reduced morbidity (Temple et al. [28]; Singh et al. [16]). Integration of SMS education with ASHA outreach would further amplify reach, particularly among first-time mothers who often lack prior experiential knowledge.

To institutionalize such programs, policy makers should adopt a three-tier model: National: develop standardized, evidence-based ANC message libraries validated by experts; Regional: translate and culturally adapt content into local languages (as done in this study); and Local: integrate delivery with district health information systems for monitoring engagement and outcomes. Such an approach would operationalize WHO’s recommendation for “digital client communication” as a routine component of maternal care (Lund et al. [17]; AlJaberi [25]).

Strengths and novel contributions

The Active Moms initiative makes a novel contribution by demonstrating the feasibility of a linguistically localized, low-cost, SMS-only health education program in a semi-urban Indian context. While many previous studies have evaluated national-level campaigns or smartphone-based applications, few have documented district-scale SMS interventions specifically targeting primigravida women. By focusing on a text-only modality, this study highlights the potential of basic mobile technology to reach populations with limited digital access and literacy.

Several methodological strengths further enhance the contribution of this study. The intervention achieved a high completion rate, with all enrolled participants completing both pre- and post-intervention assessments, indicating strong feasibility and acceptability of the SMS-based approach. The SMS content was systematically developed from WHO Antenatal Care guidelines and reviewed by domain experts, ensuring content validity. Messages were linguistically and culturally tailored to the local context of South Gujarat, improving relevance and comprehension among first-time mothers. In addition, the standardized timing and delivery of messages strengthened intervention fidelity. The inclusion of both antenatal knowledge and health attitudes as outcome measures allowed assessment of cognitive as well as affective dimensions of maternal health education, extending beyond service-utilization metrics commonly reported in mHealth studies. The use of quantitative pre-post assessment, standardized questionnaires, and effect-size reporting further adds empirical rigor that is infrequently observed in small-scale SMS interventions. Importantly, the study also advances understanding of how sociodemographic factors interact with digital health literacy, contributing context-specific insights to the existing literature (Bener et al. [29]; Bitew et al. [14]).

Limitations

This study has several limitations that should be considered when interpreting the findings. First, the relatively small sample size (N = 60) may limit statistical power and reduce the external validity of the results. Although the one-group pretest-posttest design allowed assessment of within-participant change, the absence of a control group restricts causal inference, as observed improvements cannot be attributed solely to the intervention. Additionally, this design is susceptible to testing effects, whereby improved post-intervention scores may partly reflect increased familiarity with the assessment instruments rather than true knowledge or attitude change. The study was conducted in selected hospitals in South Gujarat, which may limit the generalizability of findings to other geographic regions, healthcare settings, or cultural contexts. Furthermore, the short follow-up period captured only immediate changes in knowledge and attitudes, precluding assessment of long-term retention, sustained behavioral change, or maternal and neonatal health outcomes. Finally, reliance on self-reported measures, particularly for attitude assessment, may have introduced recall bias and social desirability bias, potentially inflating observed improvements [30]. In addition, participation was limited to women who were able to read and comprehend SMS messages, which may have introduced selection bias and limits applicability to populations with lower literacy levels.

Directions for future research

Future investigations should employ randomized controlled trials with larger, multi-site samples to establish causality and enhance generalizability. Longitudinal designs following participants through delivery would enable assessment of sustained behavior change and maternal-neonatal outcomes, including ANC attendance, anemia control, and skilled-delivery rates (Peña-Rosas et al. [31]). Comparative trials evaluating different digital formats, such as WhatsApp, interactive voice response, or hybrid SMS + app models, could identify context-specific optimal delivery channels (Sawyer et al. [32]).

Moreover, cost-effectiveness analyses and user-acceptability studies should inform large-scale deployment. Qualitative exploration of message comprehension, cultural resonance, and perceived credibility could refine content design. Integration with frontline health-worker feedback systems may convert unidirectional communication into participatory learning cycles. Such innovations would align with global trends toward personalized, data-driven mHealth ecosystems (Kamau et al. [33]; Lee et al. [34]).

## Conclusions

The Active Moms SMS-based education program was associated with improvements in antenatal knowledge and self-reported health attitudes among primigravida women in South Gujarat. These findings suggest that short, linguistically tailored text messages can serve as a practical and accessible supplement to routine antenatal counseling. The simplicity, low cost, and broad reach of SMS make this approach suitable for integration into existing maternal health services. However, improvements such as incorporating interactive messaging, assessing long-term retention, and evaluating behavior change outcomes may strengthen future interventions. Additionally, the lack of a control group may have contributed to the inflation of effect size estimates, limiting the precision of impact magnitude. Larger controlled trials with extended follow-up are recommended to validate these findings and enhance generalizability. Overall, the intervention demonstrates promising potential to strengthen maternal health literacy in resource-limited settings. These conclusions are limited to short-term changes in knowledge and self-reported attitudes, as behavioral practices and clinical outcomes were not evaluated.

## Appendices

Appendix A: Statistical computation details

Paired-Samples t-Test

Pre-intervention data were used as baseline measures for each participant to calculate within-subject differences (Δ = post - pre). These differences formed the basis for t, Cohen’s d, and η² computations.

For each participant i, the difference score is:

Di = Xpost,i - Xpre,i

where Xpre,i = pre-intervention score and Xpost,i = post-intervention score.

The mean difference (D̄) and standard deviation of the differences (SDD) are:

\[ \bar{D} = \frac{1}{n} \sum D_i \]

\[ SDD = \sqrt{\frac{\sum (D_i - \bar{D})^2}{n - 1}} \]

The paired t-statistic is computed as $t = \frac{\bar{D}}{SDD / \sqrt{n}}$, with df = n - 1. Example: If the mean difference $\bar{D}$ = 4.8, SDD = 3.5, n = 60, then t = 4.8/(3.5/$\sqrt{60}$).

Effect Size: Cohen’s d for Paired Data

Cohen’s d quantifies the magnitude of change between pre- and post-intervention scores:

\[ d = \bar{D} / SDD \]

where $\bar{D}$ = mean difference and SDD = standard deviation of the differences. Interpretation (Cohen, 1988): small ≈ 0.2, medium ≈ 0.5, large ≥ 0.8. Example: $\bar{D}$ = 4.8, SDD = 3.5 → d = 1.37 (large effect).

Partial Eta-Squared (η²)

Partial eta-squared estimates the proportion of variance in the outcome explained by the intervention:

\[ \eta^2 = \frac{t^2}{t^2 + \text{df}} \]

Interpretation (Cohen, 1988): small ≈ 0.01, medium ≈ 0.06, large ≥ 0.14. Example: t = 6.54, df = 59 → $\eta^2$ = 6.54²/(6.54² + 59) ≈ 0.42.

Pearson’s Correlation Coefficient (r)

The linear relationship between post-intervention knowledge and attitude scores was examined using Pearson’s r:

\[r = \frac{\sum (X_i - \bar{X})(Y_i - \bar{Y})}{\sqrt{\sum (X_i - \bar{X})^2} \, \sqrt{\sum (Y_i - \bar{Y})^2}} \]

 Interpretation: small ≈ 0.1, medium ≈ 0.3, large ≥ 0.5. Two-tailed significance at α = 0.05.

Chi-Square Test of Association (χ²)

Associations between categorical knowledge/attitude levels and demographic variables were analyzed using Pearson’s χ² test:

\[ \chi^2 = \sum \frac{(O_{ij} - E_{ij})^2}{E_{ij}} \]

$E_{ij}$ = (row total × column total)/grand total, df = (r - 1)(c - 1). A p-value < 0.05 indicates statistical significance.

Significance Thresholds

All tests were two-tailed. Significance was set at p < 0.05; p < 0.001 was considered highly significant. Analyses were performed using IBM SPSS Statistics version 25.0 (IBM Corp., Armonk, NY, USA).

Appendix B: Survey instruments

Structured Knowledge Questionnaire on Antenatal Care (20 Items)

Objective: To assess participants’ knowledge of essential antenatal care practices before and after the SMS-based education program (Table 6). Each correct response = 1 point; total = 20. Interpretation: Inadequate < 50%, Moderate 50%-79%, Adequate ≥ 80%.

**Table 6:** Structured Knowledge Questionnaire on antenatal care ANC: antenatal care.

Item No.	Question (English)	Response Options	Correct Answer	Domain
1	How many antenatal checkups are recommended during pregnancy?	a) 2 b) 3 c) 4 d) ≥4	d	General ANC
2	When should the first ANC visit ideally occur?	a) 1st month b) 3rd month c) 6th month d) After 7 months	b	General ANC
3	Which supplement is recommended daily during pregnancy?	a) Vitamin C b) Calcium c) Iron + Folic acid d) Multivitamin only	c	Nutrition
4	What food helps prevent anemia?	a) Rice b) Green leafy vegetables c) Butter d) Sweets	b	Nutrition
5	How many hours of sleep are advised for pregnant women?	a) 5–6 b) 6–7 c) 8–9 d) >9	c	Rest & Hygiene
6	What should be avoided during pregnancy?	a) Iron tablets b) Smoking/alcohol c) Fruits d) Exercise	b	Hygiene
7	Which sign requires immediate medical attention?	a) Mild backache b) Headache & blurred vision c) Nausea d) Appetite loss	b	Danger signs
8	How should iron tablets be taken?	a) With milk b) After meals with water c) With tea d) Before meals	b	Supplementation
9	What is the ideal spacing between two tetanus injections?	a) 1 week b) 1 month c) 6 months d) No fixed time	b	Immunization
10	What helps prevent constipation during pregnancy?	a) High-fiber foods & water b) Fasting c) Less water d) Spices	a	Nutrition
11	Handwashing before meals prevents which disease?	a) Anemia b) Diarrhea c) Cough d) Headache	b	Hygiene
12	Minimum weight gain expected during pregnancy?	a) 1–3 kg b) 4–5 kg c) 8–12 kg d) >15 kg	c	Physical changes
13	What is the normal hemoglobin level during pregnancy?	a) <9 g/dL b) ≥11 g/dL c) 10 g/dL d) 8 g/dL	b	Hematology
14	Danger signs include swelling of feet and hands.	True / False	True	Danger signs
15	Eating for two means doubling food quantity.	True / False	False	Nutrition
16	Daily walk of 20–30 minutes is recommended.	True / False	True	Physical activity
17	Antenatal checkups help detect anemia.	True / False	True	ANC purpose
18	Pregnant women should visit hospital only during labor.	True / False	False	Health-seeking
19	Stress can affect baby’s growth.	True / False	True	Psychosocial
20	Family support improves maternal health.	True / False	True	Social support

Antenatal Health Attitude Scale (22 Items)

Objective: To assess women’s attitudes toward antenatal practices after exposure to SMS education. Scale adapted and localized from Indira et al. (2017) and WHO (2016) ANC guidelines (Table 7). Response format: 3-point Likert (Agree=3, Uncertain=2, Disagree=1). Total score range = 22-66. Interpretation: Positive attitude ≥85% (≥56), Neutral 70%-84% (46-55), Negative <70% (<46).

**Table 7:** Attitude Assessment Scale for antenatal practices among primigravida women

Item No.	Statement
1	I believe regular antenatal checkups are necessary for every pregnant woman.
2	Iron and folic acid tablets should be taken daily even if they cause mild nausea.
3	Rest during the day helps the baby grow healthy.
4	Drinking plenty of water is important during pregnancy.
5	Eating vegetables and fruits daily keeps me and my baby strong.
6	Taking advice from health workers is more important than family myths.
7	I should inform a doctor if I notice swelling, headache, or bleeding.
8	Family members should help pregnant women in household work.
9	Using soap for handwashing prevents illness.
10	I feel confident to ask health questions at the clinic.
11	Walking or light exercise is good for pregnancy.
12	I feel that mobile messages can help me remember healthy habits.
13	I will avoid self-medication during pregnancy.
14	Visiting the hospital early in labor is important.
15	Taking balanced meals is my responsibility for my baby’s health.
16	Pregnant women should attend all immunization visits.
17	I believe pregnancy is a natural event but still needs medical care.
18	My husband should know about antenatal care.
19	Stress and anger are harmful for the baby.
20	SMS reminders are useful for remembering checkup dates.
21	I feel motivated to follow the advice received in SMS messages.
22	Every pregnant woman should have a delivery plan in advance.

Appendix C

Sample SMS is given in Table 8.

**Table 8:** Sample SMS SMS: short message service.

Week	Topic Focus	Number of SMS	Example Message (English translation)
1	Nutrition & Iron	5	“Eat green vegetables and one iron tablet daily to keep blood strong.”
2	Hygiene & Rest	5	“Wash hands before meals; take 8 hrs sleep for baby’s growth.”
3	Danger signs & Delivery prep	5	“If swelling or headache occurs, contact doctor immediately.”

Eat green leafy vegetables, fruits, and pulses daily during pregnancy. They help prevent anemia and keep you and your baby healthy.
